# Comparison of the Impact of Annual and Semiannual Mass Drug Administration on Lymphatic Filariasis Prevalence in Flores Island, Indonesia

**DOI:** 10.4269/ajtmh.18-0570

**Published:** 2018-12-17

**Authors:** Taniawati Supali, Yenny Djuardi, Adriani Lomiga, Sovie Nur Linda, Elisa Iskandar, Charles W. Goss, John Philip Miller, Gary J. Weil, Peter U. Fischer

**Affiliations:** 1 Department of Parasitology, Faculty of Medicine, Universitas Indonesia, Jakarta, Indonesia;; 2 Program Studi Ilmu Kesehatan Masyarakat, Program Pascasarjana, Universitas Nusa Cendana, Kupang, Indonesia;; 3 Division of Biostatistics, Washington University School of Medicine, St. Louis, Missouri;; 4 Infectious Diseases Division, Department of Medicine, Washington University School of Medicine, St. Louis, Missouri

## Abstract

We compared the impact of annual and semiannual mass drug administration (MDA) on the prevalence of *Brugia timori* and *Wuchereria bancrofti* in Flores Island. Two villages (Paga, *B. timori* only; Lewomada, co-endemic) received annual MDA with diethylcarbamazine/albendazole and a larger village (Pruda, co-endemic) received semiannual MDA. Infection parameters (microfilariae [Mf], antibodies to recombinant filarial antigen BmR1 [Brugia Rapid (BR)], and a test for *W. bancrofti* antigenemia [immunochromatographic test (ICT)]) were assessed before and after treatment. The crude Mf prevalence in Pruda decreased after five semiannual treatments from 14.2% to 1.2%, whereas the Mf prevalence in the other two villages decreased after three annual treatments from 3.9% to 0% and from 5% to 0.3%, respectively. ICT positivity prevalence in Pruda and Lewomada decreased from 22.9% and 6.5% to 7% and 0.8%, respectively, whereas BR antibody prevalence in Pruda, Lewomada, and Paga decreased from 28.9%, 31.7%, and 12.5% to 3.6%, 4.1%, and 1.8%, respectively. Logistic regression analysis indicated that that Mf, BR, and ICT prevalence decreased significantly over time and that for the Mf and ICT outcomes the semiannual treatment had higher odds of positivity. Model-adjusted prevalence estimates revealed that apparent differences in treatment effectiveness were driven by differences in baseline prevalence and that adjusted prevalence declined more rapidly in the semiannual treatment group. We conclude that in this setting, annual MDA was sufficient to reduce Mf prevalence to less than 1% in areas with low to moderate baseline prevalence. Semiannual MDA was useful for rapidly reducing Mf prevalence in an area with higher baseline endemicity.

## INTRODUCTION

Lymphatic filariasis (LF) is a common neglected tropical disease in Indonesia that may hinder economic development.^1^ The country committed to the Global Program to Eliminate Lymphatic Filariasis (GPELF) in the year 2002, but progress has been slow and variable. The national LF elimination program is based on annual mass drug administration (MDA) using a single dose of diethylcarbamazine (DEC) combined with albendazole (ALB) in all areas with microfilaremia (Mf) or antigen (*Wuchereria bancrofti*) prevalence of 1% or higher. In 2016, it was estimated that a population of about 62 million still required MDA for LF elimination. Indonesia’s population at risk ranks third in the world, behind only India and Nigeria.^2^

The epidemiology of LF in Indonesia is unique because it is caused by three different filarial species, namely *W. bancrofti*, *Brugia malayi*, and *Brugia timori*. Furthermore, *W. bancrofti* and *B. malayi* each have several distinct ecotypes with different vector species and ecology. This variability leads to varied transmission dynamics and responses to intervention.^3^ *Brugia timori* is endemic in eastern Indonesia (east of the Wallace line). *Wuchereria bancrofti* is sometimes co-endemic with *B. timori*, but it is usually transmitted by sympatric but different anopheline vector species. Previous studies by our group have shown that annual MDA can be efficiently performed in eastern Indonesia and provided evidence that LF caused by *B. timori* and *W. bancrofti* can be locally eliminated.^4,5^ Unfortunately, only 48 of the 235 evaluation units in Indonesia have passed transmission assessment surveys (TAS) and stopped MDA, whereas 80% of the implementation units still require MDA.^2^ Therefore, cost-effective strategies to accelerate LF elimination are highly desirable.

Previous modeling studies based on data from Ghana and India predicted that semiannual MDA would significantly speed up LF elimination and reduce the overall cost of MDA programs by 11–18%.^6^ To test this hypothesis for Indonesia, we compared the impact of annual and semiannual MDA with DEC and ALB on filarial infection parameters in selected areas of Flores Island where *B. timori* and *W. bancrofti* are endemic. We found that both treatment regimens were effective for dramatically reducing infection markers with no marked advantage of twice-yearly MDA.

## METHODS

### Ethical approval and selection of the study area.

The study received ethical approval from the Ethics Committee of Faculty of Medicine, University of Indonesia (No.61/PT02.FK/ETIK/2011). This study was registered at ClinicalTrials.gov with identifier No NCT01905423.

The study was initiated in 2011 with screening surveys to identify and select LF-endemic villages in Sikka, Flores Timur, and Lembata districts in the province East Nusa Tenggara Timur. In these surveys Mf were detected by thick night blood smears. In Flores Timur, a convenience sample of 2,584 subjects residing in 24 villages was tested and eight villages were Mf positive with crude prevalence between 1% and 16%. One village was co-endemic for *B. timori* and *W. bancrofti*, whereas all other villages were endemic for *W. bancrofti* only. In Lembata, we screened 653 subjects from seven villages, and only nine *W. bancrofti* Mf-positive individuals were identified in a single village. In Sikka district, 2,341 subjects from 17 villages were tested for Mf, and 16 villages were positive for *B. timori* with crude Mf prevalence of 1–22%. In addition, three villages were co-endemic for *W. bancrofti*. Because we wanted to focus on brugian filariasis, we selected three villages in Sikka district for our study.

### Study area and population.

**Figure 1.**
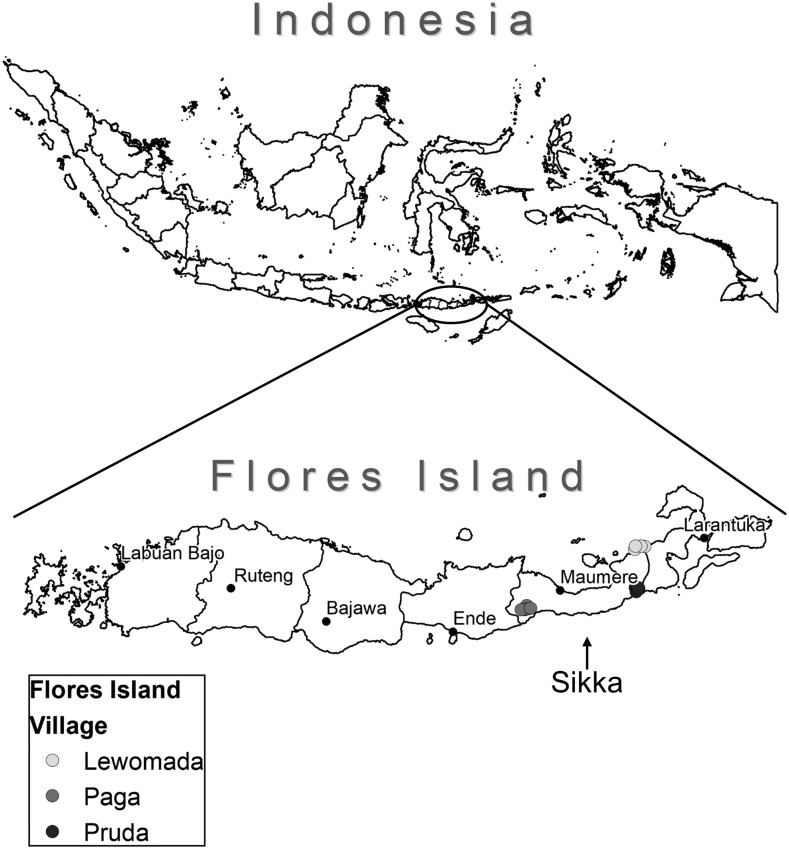
Map of Indonesia and Flores Island showing the study sites in Sikka district. Pruda received semiannual mass drug administration (MDA), whereas Lewomada and Paga received annual MDA.

Sikka district (population approximately 300,000 in 2010) is located on Flores Island (Figure 1). The district comprises 21 sub-districts with 147 villages that are served by 22 primary health centers (Puskesmas). With the exception of the district capital Maumere, most areas are rural and often served only by dirt roads or boat (http://www.sikkakab.go.id). The study village Paga is in the southwestern part of Sikka, whereas Pruda and Lewomada are in the more remote eastern part of the district (Figure 1).

Four cross-sectional field surveys were performed in the study villages, at baseline before MDA and at 12 months, 24 months, and 36 months after the initial MDA. During the survey, all eligible individuals aged 5 years and older who consented to the study provided demographic information (gender, age, and bed net use) and were tested for Mf, antifilarial antibodies, and circulating filarial antigen. Individuals who were not eligible for MDA or who moved to the village within the last year were not included in the study. Individuals who did not consent to the survey but were eligible and consenting with MDA were offered MDA participation only.

### Treatment.

Three villages were assigned to two MDA groups. The first group (Paga and Lewomada) received annual treatment with DEC (6 mg/kg) combined with ALB (400 mg) and Pruda village received twice-yearly treatment with DEC/ALB. The distribution of DEC/ALB was performed in collaboration with the health authority of the local government and the Puskesmas. The first rounds of MDA were distributed to all eligible residents in Paga and Pruda in March 2011 and in Lewomada in March 2012. Annual MDA was provided every 12 months, whereas Pruda received additional treatments at month 6 and 18 after the first treatment. Thus, Paga and Lewomada received a total of three rounds of MDA over a period of 24 months, whereas Pruda received five rounds. Reexaminations were performed annually, and the last survey was performed 36 months after the first treatment (12 months after the last round of MDA). The last surveys were performed in March 2014, in two study villages (Paga and Pruda), and in March 2015 (Lewomada).

### Detection of Mf by microscopy.

A total of 250 μL of finger blood was collected at night (between 8 and 11 pm) in an ethylenediaminetetraacetic acid (EDTA)-coated tube. Sixty microliters of blood was evenly spread in three lines onto a clean glass slide labeled with unique barcode for each participant, dried for 2 days, de-hemoglobinized for 3 minutes, air-dried, fixed with methanol for 1 minute, and stained with Giemsa (1:14) for 15 minutes. The stained slides were examined under the microscope for the presence of Mf. The result from each slide was entered into a cellular phone and linked to the participant’s barcode with a barcode reader. The remaining blood samples were centrifuged, plasma was separated for later immunoglobulin G4 (IgG4) antibody or antigen detection, and stored at −20°C. Plasma samples were transported with ice packs to the laboratory of the Department of Parasitology in Jakarta and stored at −20°C until use.

### Detection of antifilarial antibodies and circulating filarial antigen.

Specific antifilarial IgG4 antibodies reactive to the recombinant *B. malayi* antigen BmR1 were detected using the Brugia Rapid test (BR) (Reszon Sdn Bhd, Bangi, Malaysia). The test was performed using 25 µL of plasma according to the recommendation of the manufacturer.^5^ Circulating *W. bancrofti* antigen was detected with the Binax Now^®^ Filariasis Test (ICT) (Alere, Scarborough, ME) with stored plasma according to the protocol provided by the manufacturer. The result was read strictly at 10 minutes after application of the sample.

### Data management and analysis.

All data were recorded and synchronized in a Motorola cellular phone (XT 720; Motorola, Chicago, IL) before being sent to the server using the LinksSystem.^7^ For analysis, the data were downloaded as Excel files from the server and transferred into SPSS version 20 (Armonk, NY) or SAS version 9.4 (Cary, NC). The data before and after treatment were regarded as independent data, so unpaired statistical tests were used. The unadjusted Mf prevalence and antibody and antigen prevalence in each village before and after treatment were compared and analyzed using a chi-square test. Changes in infection prevalence of filarial infections were assessed by Fisher’s exact test. The geometric mean of Mf density was calculated with data from persons with microfilaremia. The Mf, ICT, and BR outcomes were later analyzed using a logistic regression analysis; neighborhood nested within village was treated as a random effect to account for correlation among subjects within a neighborhood (PROC GLIMMIX, SAS 9.4). In this analysis, we assessed the treatment regimen, time, and their interaction. The interaction term was retained if it was significant (*P* < 0.05), otherwise it was removed from the model. The final model for each outcome was also used to obtain model-adjusted prevalence estimates. Two of the villages did not have BR data for years 1 and 2. Thus, only the baseline and 3-year data were considered in the analysis of this outcome.

## RESULTS

### Demographics and treatment coverage.

The number of subjects enrolled each year varied between 2,804 and 3,198 subjects (Table 1). In all villages, fewer males (between 37.8% and 47.9%) were examined compared with females. Parameters such as age distribution and bed net use were very similar between the two treatment areas. Compliance (subjects who reported taking antifilarial drugs during MDA) was assessed during the follow-up surveys. This varied between 70.1% and 89.8% with the highest reported compliance observed in Pruda, the area that received twice-yearly MDA (Table 2).

**Table 1:** Examined study population in the three sentinel study areas in Sikka district, province Nusa Tenggara Timur, Indonesia

	Paga		Lewomada		Pruda		Total	
	Males (%)	Total	Males (%)	Total	Males (%)	Total	Males (%)	Total
Pre-mass drug administration	546 (37.8)	1,443	311 (43.1)	722	488 (47.2)	1,033	1,345 (42.1)	3,198
Year 1	392 (39.4)	994	360 (42.6)	846	475 (47.1)	1,007	1,227 (43.1)	2,847
Year 2	426 (38.2)	1,114	382 (44.5)	858	500 (47.9)	1,043	1,308 (43.4)	3,015
Year 3	358 (39.6)	905	400 (45.9)	871	437 (42.5)	1,028	1,195 (42.6)	2,804

**Table 2:** Compliance with MDA in the study population (aged 5 years and older) in the three sentinel study areas in Sikka district

	Paga		Lewomada		Pruda		Total	
	*N*	%	*N*	%	*N*	%	*N*	%
Year 1	735	73.9	683	80.7	817	81.1	2,235	78.5
Year 2	781	70.1	614	71.6	847	81.2	2,242	74.4
Year 3	731	80.8	648	74.4	922	89.8	2,301	82.1

MDA = mass drug administration. Participants were asked during the survey whether they participated in any lymphatic filariasis MDA in the previous year.

### Prevalence and density of Mf.

During the pre-MDA surveys, it became clear that the baseline prevalence in the three study areas (Paga, Lewomada, and Pruda) was more different than expected: Whereas Paga and Lewomada had a low unadjusted Mf prevalence of 3.9% and 5.0%, respectively; the Mf prevalence in Pruda was 14.2% (Table 3). In Paga, the low prevalence was relatively evenly distributed, whereas in Lewomada, most of the area had low prevalence, but one sub-village (Henga) with about 300 residents had a higher prevalence of about 10%.

**Table 3:** Summary of prevalence of Mf, positive BR IgG4 antibody test, and circulating *Wuchereria bancrofti* antigen (ICT) before MDA and following 1, 2, and 3 years of initiating MDA

	Paga			Lewomada			Pruda		
	% Mf (CI)	% BR (CI)	% ICT (CI)	% Mf (CI)	% BR (CI)	% ICT (CI)	% Mf (CI)	% BR (CI)	% ICT (CI)
Pre-MDA	3.9 (3.0–5.0)	12.5 (10.5–14.7)	Nd	5.0 (3.6–6.8)	31.7 (28.3–35.2)	6.5 (4.9–8.5)	14.2 (12.2–16.5)	28.9 (25.7–32.3)	22.9 (20.4–25.5)
Year 1	1.1 (0.6–2.0)	Nd	Nd	1.1 (0.6–2.0)	19.9 (17.2–22.8)	1.5 (0.8–2.5)	3.8 (2.7–5.1)	Nd	9.1 (7.4–11.0)
Year 2	0.5 (0.2–1.2)	Nd	Nd	0.7 (0.3–1.5)	15.7 (13.4–18.3)	1.5 (0.8–2.5)	1.4 (0.9–2.4)	Nd	10.2 (8.5–12.3)
Year 3	0 (0.0–0.4)	1.8 (1.1–2.9)	Nd	0.3 (0.1–1.0)	4.1 (2.9–5.6)	0.8 (0.4–1.7)	1.2 (0.7–2.0)	3.6 (2.6–5.0)	7.0 (5.6–8.8)

BR = Brugia Rapid; CI = 95% confidence interval; MDA = mass drug administration; Mf = microfilariae; Nd = not determined. Paga and Lewomada received annual MDA (three rounds), whereas Pruda received semiannual MDA (five rounds).

Following three rounds of MDA, Mf prevalence decreased to zero in Paga and to 0.3% in Lewomada, whereas after five semiannual rounds of MDA, the Mf prevalence in Pruda was still 1.2% (Table 3). Logistic regression analysis of Mf results showed that the overall effect for the semiannual treatment regimen had 3.9 times higher odds (*P* < 0.001) of having Mf compared with subjects receiving an annual dosage (Table 4). This difference is likely driven by the higher overall prevalence differences at baseline in the different villages (see the previous paragraph). Although there was not a significant treatment × time interaction, there was an overall significant decrease in Mf over time (*P* < 0.001), indicating that the odds of Mf positivity decreased by 67% each year. Furthermore, model-adjusted prevalence estimates indicated that the twice-yearly treatment decreased the adjusted Mf prevalence more rapidly through time as compared with the annual treatment regimen (Table 5).

**Table 4:** Logistic regression analysis of major infection parameter outcome

Outcome	Variable	Group	Adjusted odds ratio (95% confidence interval)	*P*-value
Microfilariae	Treatment	1 Dose/year	1.0	< 0.001
		2 Doses/year	3.92 (1.98, 7.78)	
	Visit	NA	0.33 (0.28, 0.39)	< 0.001
Brugia Rapid	Treatment	1 Dose/year	1.0	0.31
		2 Doses/year	1.49 (0.67, 3.31)	
	Visit	NA	0.08 (0.06, 0.11)	< 0.001
ICT antigen*	Treatment	1 Dose/year	1.0	< 0.001
		2 Doses/year	14.24 (6.2, 32.69)	
	Visit	Slope 1 dose/year	0.45 (0.35, 0.58)	< 0.001
		Slope 2 doses/year	0.62 (0.56, 0.69)	< 0.001

Outcome was adjusted for potential correlation among subjects within a neighborhood. Neighborhood (village) was treated as a random effect for all villages.

* The Treatment × visit interaction effect for this outcome was significant (*P* = 0.016), and the odds ratios of the slopes for annual and semiannual treatment are reported to facilitate interpretation.

**Table 5:** Model predictions (adjusted prevalence) of major infection parameter outcome for annual (1 dose/year) and semiannual (2 doses/year) mass drug administration

Treatment regimen	Round	Model-adjusted prevalence and 95% confidence interval		
		Mf	BR	ICT
1 Dose/year	Baseline	3.39 (2.12, 5.37)	19.8 (12.77, 29.4)	1.83 (1, 3.32)
2 Doses/year	Baseline	12.09 (7.57, 18.75)	26.91 (16.45, 40.77)	20.94 (13.09, 31.78)
1 Dose/year	1 year	1.16 (0.73, 1.83)	ND	0.83 (0.46, 1.47)
2 Doses/year	1 year	4.39 (2.7, 7.05)	ND	14.18 (8.67, 22.34)
1 Dose/year	2 year	0.39 (0.24, 0.64)	ND	0.37 (0.2, 0.71)
2 Doses/year	2 year	1.51 (0.89, 2.53)	ND	9.35 (5.58, 15.25)
1 Dose/year	3 year	0.13 (0.07, 0.23)	2.04 (1.22, 3.4)	0.17 (0.08, 0.37)
2 Doses/year	3 year	0.51 (0.28, 0.92)	3.02 (1.63, 5.52)	6.04 (3.5, 10.24)

Outcome was adjusted for potential correlation among subjects within a neighborhood. Neighborhood (Village) was treated as a random effect for all villages.

**Figure 2.**
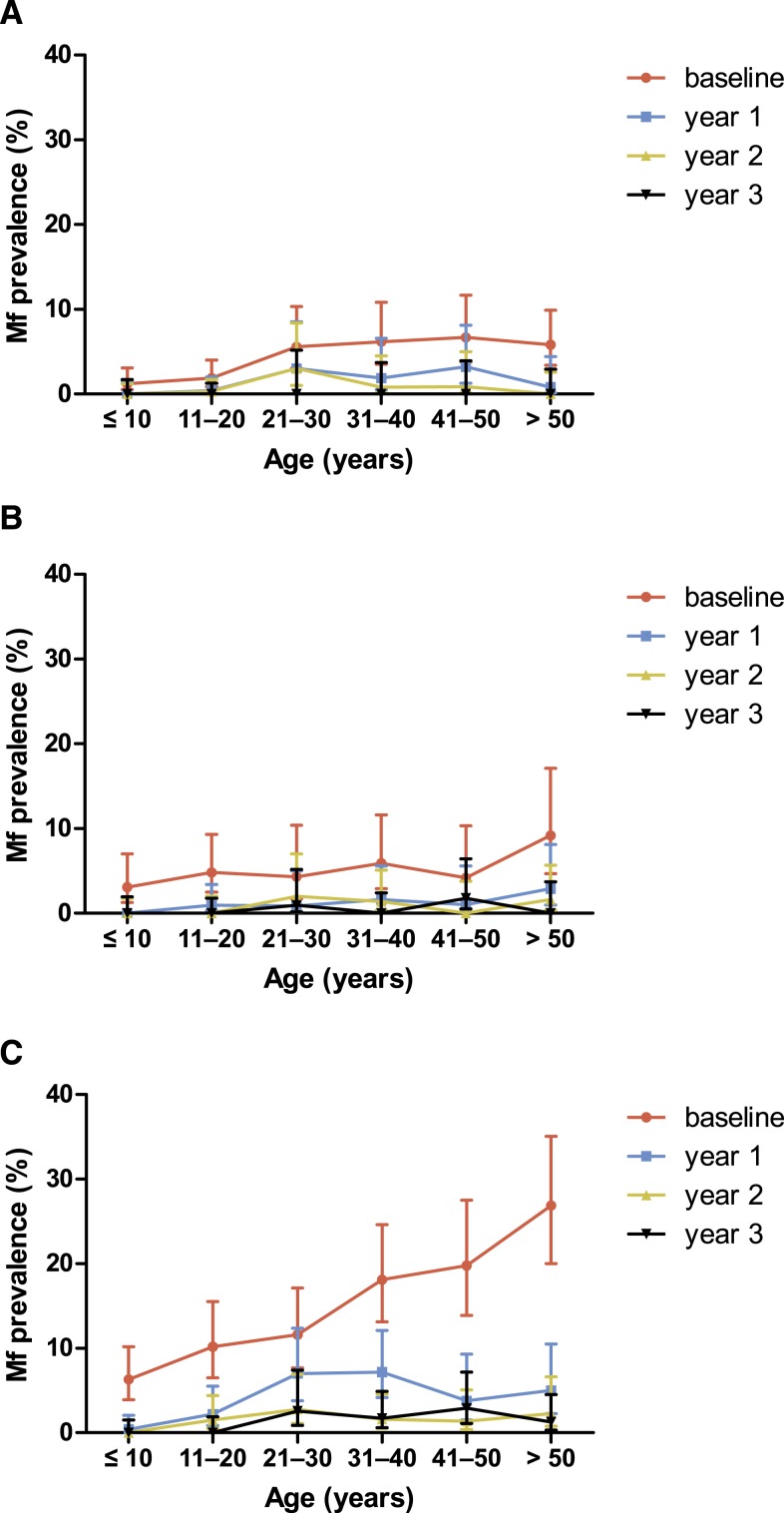
Microfilariae (Mf) prevalence in the study villages Paga (**A**), Lewomada (**B**), and Pruda (**C**) by age group, at baseline and follow-up 1, 2, and 3 years following initiation of mass drug administration (MDA). Paga and Lewomada received three rounds of MDA after the baseline survey and at 12 and 24 months, whereas Pruda received five rounds of MDA with additional rounds at 6 and 18 months. This figure appears in color at www.ajtmh.org.

Analysis of Mf prevalence by age group showed similar patterns in all three study areas with low prevalence in the younger participants and higher prevalence in older participants (Figure 2). In all areas, reduction in Mf prevalence after MDA was also higher in younger subjects.

The Mf prevalences at baseline in males in Paga, Lewomada, and Pruda were 4.4% (geometric mean in positives, 172 Mf/mL), 6.4% (geometric mean 517 Mf/mL), and 18.2% (geometric mean 284 Mf/mL), respectively. Corresponding prevalence in females were 3.6% (geometric mean 104 Mf/mL), 3.9% (geometric mean 160 Mf/mL), and 10.7% (geometric mean 178 Mf/mL), respectively (Table 6). The gender differences for prevalence and density were more dramatic for participants older than 14 years. After three or five rounds of MDA in total, only three Mf-positive adult men but 12 Mf-positive adult women were detected. This suggests that after MDA, women of child-bearing age may still represent a reservoir for Mf.

**Table 6:** Comparison of the Mf prevalence and density in infected male and female subjects by village and year

Village	Age (years)	Gender	Baseline			Year 1			Year 2			Year 3		
			*N*	Mf+ (*n*, %)	Geomean (Mf/mL)	*N*	Mf+ (*n*, %)	Geomean (Mf/mL)	*N*	Mf+ (*n*, %)	Geomean (Mf/mL)	*N*	Mf+ (*n*, %)	Geomean (Mf/mL)
Paga	≤ 15	Male	289	4 (1.4)	301	220	0 (0)	0	248	0 (0)	0	197	0 (0)	0
		Female	308	5 (1.6)	132	251	0 (0)	0	294	0 (0)	0	232	0 (0)	0
	> 15	Male	239	19 (7.9)	153	172	9 (5.2)	103	178	2 (1.1)	240	160	0 (0)	0
		Female	555	26 (4.7)	99	351	2 (0.6)	171	394	4 (1.0)	95	313	0 (0)	0
	All	Male	528	23 (4.4)	172	392	9 (2.3)	103	426	2 (0.5)	240	357	0 (0)	0
		Female	863	31 (3.6)	104	602	2 (0.3)	171	688	4 (0.6)	95	545	0 (0)	0
Lewomada	≤ 15	Male	144	7 (4.9)	374	175	2 (1.1)	82	174	0 (0)	0	174	0 (0)	0
		Female	141	6 (4.3)	229	170	0 (0)	0	177	0 (0)	0	168	0 (0)	0
	> 15	Male	167	13 (7.8)	615	185	6 (3.2)	52	203	5 (2.4)	93	224	1 (0.4)	17
		Female	270	10 (3.7)	128	316	1 (0.3)	50	299	1 (0.3)	50	303	2 (0.7)	306
	All	Male	311	20 (6.4)	517	360	8 (2.2)	58	382	5 (1.3)	93	398	1 (0.3)	17
		Female	411	16 (3.9)	160	486	1 (0.2)	50	476	1 (0.2)	50	471	2 (0.4)	306
Pruda	≤ 15	Male	200	17 (8.5)	151	207	1 (0.5)	6,383	200	1 (0.5)	350	203	0 (0)	0
		Female	174	11 (6.3)	164	192	2 (1.0)	47	194	1 (0.5)	183	195	0 (0)	0
	> 15	Male	284	71 (25.0)	330	261	20 (7.7)	232	300	6 (2.0)	84	234	2 (0.9)	33
		Female	369	47 (12.7)	181	322	14 (4.3)	77	349	7 (2.0)	121	395	10 (2.5)	51
	All	Male	484	88 (18.2)	284	468	21 (4.5)	271	500	7 (1.4)	103	437	2 (0.5)	33
		Female	543	58 (10.7)	178	514	16 (3.1)	73	543	8 (1.5)	127	590	10 (1.7)	51

Mf = microfilariae.

### *Brugia timori* and *W. bancrofti* coinfections.

No *W. bancrofti* Mf were detected at baseline or during follow-up surveys in Paga. Furthermore, the prevalence of circulating *W. bancrofti* antigen as measured by ICT was less than 1% at baseline. Therefore, this area was considered to be not endemic for *W. bancrofti* and not further tested by ICT. By contrast, Lewomada and Pruda areas were both co-endemic for *B. timori* and *W. bancrofti*. In Lewomada (at baseline), 36 Mf-positive subjects were identified (5% of the total tested, including 13 children younger than 16 years): 33 were positive for *B. timori* only with a geometric mean density of 290 Mf/mL, two individuals were Mf positive for *B. timori* (33 Mf/mL and 867 Mf/mL, respectively) and *W. bancrofti* (1,383 Mf/mL and 1,067 Mf/mL, respectively), and one individual was Mf positive for *W. bancrofti* only (67 Mf/mL). All three *W. bancrofti* Mf-positive subjects were also ICT positive.

Mf prevalence was highest in Pruda. Among the 146 Mf-positive subjects (14.2% of the total tested), 106 (10.3%) were *B. timori* Mf positive and 89 (8.6%) were *W. bancrofti* Mf positive. Among the *W. bancrofti* Mf positives, 87 (97.8%) were also ICT positive. Based on the prevalence of single infections and under the assumption of independent transmission, it was calculated that prevalence of coinfections should be about 0.09%. Interestingly, the number of Mf-positive coinfections was 49 (4.8% of the total number of participants tested) and much higher than expected (Table 7). Because of this fairly even distribution of both filarial infections, we were able to assess the effect of MDA on both species in the same study area. The Mf prevalence of *B. timori* only, *W. bancrofti* only, and coinfections in this area decreased from 5.6%, 3.9%, and 4.8% at baseline to 1.0%, 0.2%, and 0% after five rounds of MDA, respectively (Table 7).

**Table 7:** The change of Mf prevalence and density over time in single and in mixed infection of *Brugia timori* and *Wuchereria bancrofti* in Pruda, the twice-yearly treated village

	*N*	*B. timori*			*W. bancrofti*			*B. timori* + *W. bancrofti*			
		*n*	Mf+ (%, CI)	Geomean (Mf/mL)	*n*	Mf+ (%, CI)	Geomean (Mf/mL)	*n*	Mf+ (%, CI)	Geomean *B. timori* (Mf/mL)	Geomean *W. bancrofti* (Mf/mL)
Pre-mass drug administration	1,027	57	5.6 (4.3–7.1)	114 (77–167)	40	3.9 (2.9–5.3)	148 (97–227)	49	4.8 (3.6–6.2)	401 (251 –641 )	163 (106–249)
Year 1	982	10	1.0 (0.5–1.9)	200 (40–1,008)	23	2.3 (1.6–3.5)	105 (64–170)	4	0.4 (0.2–1.0)	297 (83–1,070)	157 (9–2,645)
Year 2	1,042	5	0.5 (0.2–1.1)	120 (23–632)	6	0.6 (0.3–1.2)	102 (23–453)	3	0.3 (0.1–0.8)	82 (7–1,045)	126 (18–871)
Year 3	1,027	10	1.0 (0.5–1.8)	54 (25–119)	2	0.2 (0.05–0.7)	24 (0.3–1,927)	0	0 (0.0–0.4)	Nd	Nd

CI = confidence interval; Mf = microfilariae.

### Prevalence of positive Brugia Rapid antibody tests.

The unadjusted prevalence of filaria-specific IgG4 antibodies as assessed by the BR test was 12.5% at baseline in the once-yearly treatment areas Paga and Lewomada and 41.7% in Pruda (Table 3). At the reexamination 3 years after the first round of MDA, BR prevalence decreased significantly to 1.8% and 4.1% in the annual and semiannual treatment areas, respectively. Logistic regression analysis showed no significant difference between changes in BR prevalence by treatment area (Table 4). BR prevalence decreased by 90% over 3 years in both areas (Table 4).

**Figure 3.**
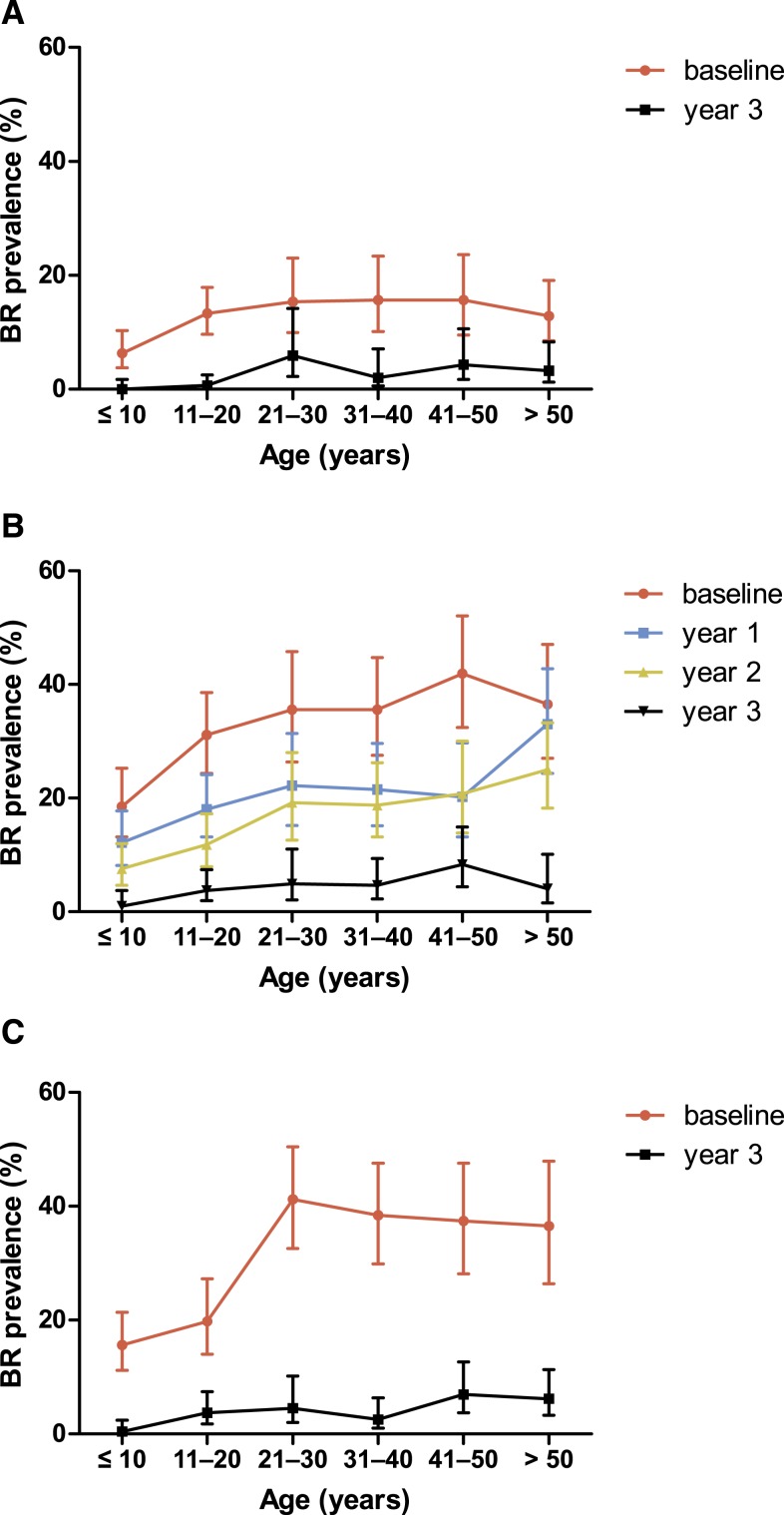
Prevalence of positive Brugia Rapid (BR) antibody tests in the study villages Paga (**A**), Lewomada (**B**), and Pruda (**C**) by age group at baseline and 3 years following initiation of mass drug administration (MDA). In addition, in Lewomada Brugia Rapid tests were also performed in year 1 and 2 after the start of MDA. Paga and Lewomada received three rounds of MDA after the baseline survey and at 12 and 24 months, whereas Pruda received five rounds of MDA with additional rounds at 6 and 18 months. This figure appears in color at www.ajtmh.org.

Analysis of BR antibody prevalence in the annual MDA villages by age shows that almost no children and young adults aged 20 years and younger were positive in Paga, whereas more children and young adults were positive in Lewomada (Figure 3A and B). Although the Mf prevalence was highest in Pruda, it was a *B. timori*/*W. bancrofti* mixed infection area, and the baseline BR prevalence was only 28.9% (lower than in Lewomada). The BR prevalence decreased to 3.6% in year 3 (Table 3). As in Lewomada, few children and young adults were BR positive in year 3. These results show that in all villages, the BR prevalence decreased significantly in all age groups after MDA (Figure 3A–C). Thus, BR prevalence decreased relatively quickly in response to MDA following the clearance of Mf.

### ICT antigen test results.

*Wuchereria bancrofti* Mf-positive individuals were only detected in Lewomada and Pruda. Baseline ICT antigen prevalences for these areas were 6.5% and 22.9%, respectively. After three rounds of MDA in Lewomada and five rounds of MDA in Pruda, ICT prevalences decreased to 0.8% and 7.0%, respectively (Table 3). The decrease in antigen prevalence in Lewomada with a low baseline antigen prevalence was more rapid than that in Pruda where the baseline prevalence was high. Logistic regression analysis of the posttreatment ICT results showed that the semiannual treatment had higher odds of ICT positivity as compared with the annual treatment group. The interaction was significant and the slope estimates indicated that the annual treatment group had a stronger decline through time when compared with the semiannual treatment group (Table 4). However, model-adjusted prevalence estimates revealed that there is a stronger decline between baseline and 3-year in the semiannual treatment group (20.9% at baseline to 6% at 3 years) as compared with the annual treatment group (1.8% at baseline to 0.17% at 3 years) (Table 5). Analysis of results from both Mf and ICT outcomes suggests that interpretation of effects on the odds scale masks the more rapid changes in prevalence through time for the semiannual treatment group that is evident in the unadjusted and adjusted prevalence estimates.

**Figure 4.**
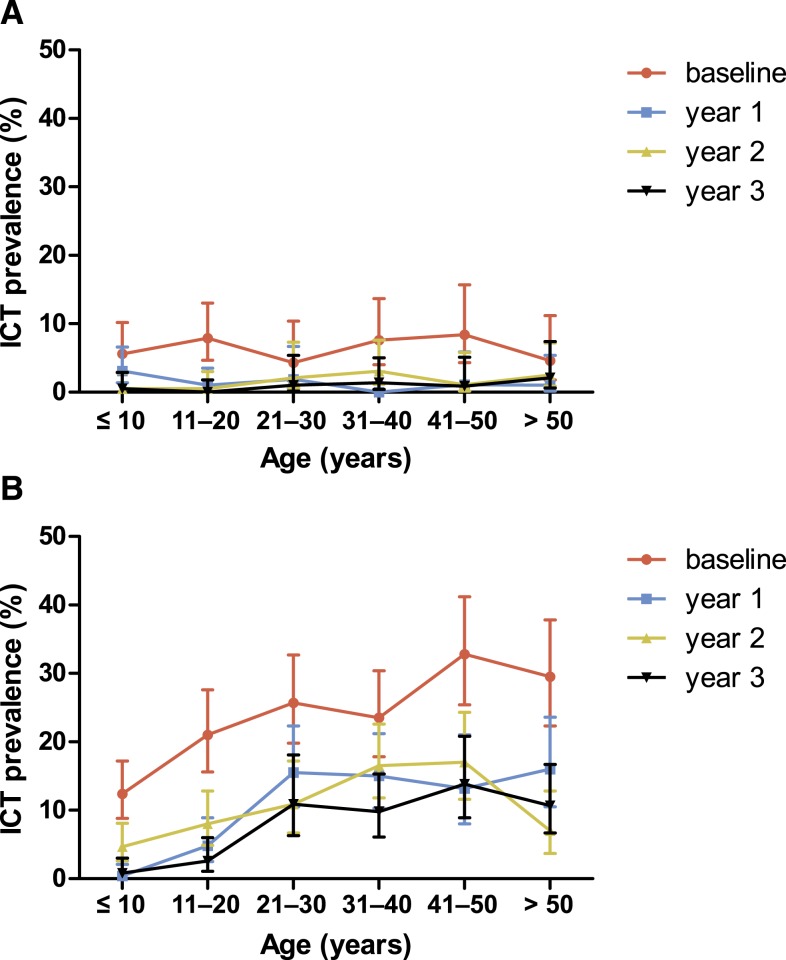
Prevalence of positive ICT antigen tests for *Wuchereria bancrofti* in the study villages Lewomada (**A**) and Pruda (**B**) by age group, at baseline and follow-up 1, 2, and 3 years following initiation of mass drug administration (MDA). Lewomada received three rounds of MDA after the baseline survey and at 12 and 24 months, whereas Pruda received five rounds of MDA with additional rounds at 6 and 18 months. This figure appears in color at www.ajtmh.org.

Analysis of ICT antigen prevalence by age group is consistent with this finding (Figure 4). It appears that *W. bancrofti* antigen is cleared faster in persons with light infections (in low prevalence areas or in younger subjects).

## DISCUSSION

Indonesia has a 50-year history of local LF control using village-based low-dose DEC distribution. A classic example was the sustainable control of *B. timori* in a single village in Flores Island.^8,9^ Unfortunately, elimination of LF was not the goal of this intervention, efforts were restricted to relatively small geographic areas, and the fear of adverse events, especially in areas with brugian filariasis, hindered the expansion of the efforts. After clinical trials indicated that DEC combined with ALB was safe and efficacious for LF caused by *B. timori*,^10,11^ Indonesia joined the GPELF in 2002 and implemented the WHO recommended MDA strategy using annual administration of this drug combination.^12^ In the present study, we compared the effect of three annual rounds or five semiannual rounds of MDA using DEC combined with ALB on LF caused by *B. timori* and *W. bancrofti* in Flores Island, Indonesia. Reported compliance rates were above 65% in all study areas, and it appears that semiannual MDA did not significantly increase the cumulative compliance as reported by study participants.

The baseline LF prevalences in the study areas were lower in areas that received three rounds of annual MDA, whereas the higher prevalence area received five rounds of semiannual MDA. In the two areas that received annual MDA, both the unadjusted and adjusted Mf prevalence decreased after two rounds of MDA to below 1%. This threshold indicates in pre-TAS that the area is eligible for TAS.^13^ In the area with higher baseline prevalence (Pruda), the unadjusted prevalence still exceeded this threshold after five semi-annual rounds of MDA, suggesting that additional rounds are needed. These results indicate that baseline Mf prevalence may be a more important determinant for whether an area is ready for TAS than the number of rounds or frequency of MDA alone. Many studies have shown that high compliance is a key factor for a successful MDA program.^6,14^ Low prevalence areas with good compliance may be eligible for TAS even after a couple of rounds, whereas some high prevalence areas may need more than five rounds of semiannual MDA despite good compliance.

At baseline, we observed higher Mf prevalence in males compared with females, especially in adults older than 15 years, and this subpopulation should be specifically addressed during social mobilization in early stages of MDA programs. Higher Mf prevalence in men is often seen in LF.^15^ Interestingly, at the end of the MDA program, most remaining Mf-positive subjects were women older than 15 years. Therefore, it may be helpful to specifically target adult women in eastern Indonesia in later stages of LF elimination programs. Previous studies in eastern Indonesia on the knowledge, attitudes, and practices concerning LF have indicated that factors that influence compliance change over the course of an LF MDA program, and social mobilization campaigns may have to be specifically tailored to changing target groups.^16^

Differences in baseline prevalence make it difficult to compare the impact of annual and semiannual MDA in the present study. Computer modeling studies using assumptions for *W. bancrofti* infections from Ghana and India predicted that twice-yearly MDA would reduce the duration of MDA programs in half and reduce the overall program costs.^6^ Although this may be true in some settings, our data from Flores are not consistent with these predictions. It appears that annual MDA with DEC and ALB can be highly effective in areas with low to moderate Mf prevalence, and high compliance may be more important than the frequency of MDA rounds.

Whereas the Paga area was endemic for *B. timori* alone, the other two areas were co-endemic for *B. timori* and *W. bancrofti*. The prevalence of circulating *W. bancrofti* antigenemia in Lewomada was 6.5%, but only three *W. bancrofti* Mf-positive individuals were detected at baseline. Therefore, we focused our comparison of the effects of MDA on the two infections on the data from Pruda, which had high baseline prevalences for both infections. Our results showed clearly that at baseline, coinfections with *B. timori* and *W. bancrofti* were more common than expected. Both filarial species are transmitted by anopheline mosquitos, but by different vector species. On Alor Island, for example, *B. timori* is transmitted by *Anopheles barbirostris*, whereas *W. bancrofti* is transmitted by *Anopheles subpictus*.^15^ The high prevalence of coinfection suggests that the same people may be heavily exposed to bites from both vector mosquito species. Furthermore, it appears that both filarial species responded well to MDA; because the prevalence of *B. timori* was higher than that of *W. bancrofti* at baseline, the residual prevalence of *B. timori* was also slightly higher after five rounds of MDA.

In the absence of a reliable antigen test for brugian filariasis, the BR test has been recommended for mapping and monitoring and evaluation of LF elimination programs in *Brugia*-endemic areas.^13^ Previous studies have shown that this antibody test is sensitive for both *B. malayi* and *B. timori* infections^17^ and that BR prevalences decrease following MDA.^5^ However, the present study demonstrates that BR prevalences decreased rapidly after MDA in three different areas: in the low prevalence area (Paga), the prevalence decreased over 36 months to 14.4% of the baseline prevalence and in the higher prevalence areas, it decreased to 12.9% (Lewomada) and 12.5% (Pruda) of the baseline values. Furthermore, the analysis by age group showed that the decline was seen in all age groups and was not limited to children and young adults. The posttreatment decrease in BR antibody prevalence is faster than that reported from *W. bancrofti* infections for antibodies reactive to the filarial antigen Bm14.^18^ This may be related to a special property of recombinant BmR1 antigen used in the test and to the diagnostic platform. In previous studies, antibodies reactive with Bm14 antigen were detected by ELISA, whereas the point-of-care BR test used in the present study detects antibodies reactive with BmR1 antigen by paper immunochromatography. IgG4 antibodies reactive with the BmR1 antigen may decrease quickly below the detection threshold of BR, but this does not necessarily mean that antifilarial antibodies reactive with the BmR1 antigen have been totally cleared.

The areas Lewomada and Pruda were co-endemic for *W. bancrofti*, and circulating antigen prevalences were assessed by ICT. ICT prevalences decreased over 36 months to 12.3% and 30.6% of the baseline in these two treatment areas, respectively. The more rapid decrease in Lewomada may be related to the relatively low prevalence and intensity of infection in that treatment area. Decreases in ICT prevalence following MDA have been reported many times before, and it is well known that circulating antigen levels decrease a few months after successful treatment.^19,20^ The finding that BR prevalence decreased more rapidly than ICT in Pruda (co-endemic for *B. timori* and *W. bancrofti*) is an interesting new finding.

In conclusion, our study did not detect an obvious advantage of twice-yearly MDA in areas that were co-endemic for *B. timori* and *W. bancrofti*. However, differences in baseline infection prevalence in this study may have obscured the potential superiority of semiannual MDA. The study showed that three rounds of once-yearly MDA with fairly high compliance reduced Mf prevalence to below 1% in low-endemicity areas. High compliance with annual MDA may be sufficient to eliminate LF in most settings, and compliance is probably more important than the frequency of MDA. Our results also suggest that the BR antibody test is a good marker for successful MDA in areas with brugian filariasis.
